# Supplementary material for: SARS-CoV-2 detection and genomic sequencing from hospital surface samples collected at UC Davis
Source: PLoS One. 2021 Jun 24;16(6):e0253578. doi: 10.1371/journal.pone.0253578 (PMC8224861; doi:10.1371/journal.pone.0253578)
Supplement: S1 Table — “U” is Undetermined (at 45 cycles of qRT-PCR). All patient rooms were occupied by known COVID-19 cases. The 1st wave was in the spring of 2020, and the second was in late summer 2020. (DOCX) [file pone.0253578.s001.docx]

S1 Table

| **Date** | **Ct (N1)** | **Ct (N2)** | **Location** | **Surface** |
| --- | --- | --- | --- | --- |
| 4/25/2020 | 36 | 39 | Patient room D14 | Floor |
| 4/25/2020 | 32 | 34 | Patient room D14 | Floor |
| 4/25/2020 | 37 | U | Patient room T7 Blue | Vent tubing arm |
| 4/25/2020 | U | 38 | Patient room T7 Blue | Keyboard |
| 4/25/2020 | 37 | 39 | Patient room T7 Blue | Telemetry screen alarm button |
| 4/25/2020 | 36 | 37 | Patient room T7 Blue | Soiled linen lid |
| 8/4/2020 | 39 | 41 | Floor Samples | Women's bathroom |
| 8/14/2020 | 35 | 37 | ICU Patient Room | Linen Cart- lid |
| 8/14/2020 | U | 42 | ICU Patient Room | Room Door Handle |
| 8/14/2020 | 38 | U | Floor Nursing Workspace | Floor |
| 8/14/2020 | 44 | U | Floor Patient Room | Floor |
