# Supplementary material for: SARS-CoV-2 detection and genomic sequencing from hospital surface samples collected at UC Davis
Source: PLoS One. 2021 Jun 24;16(6):e0253578. doi: 10.1371/journal.pone.0253578 (PMC8224861; doi:10.1371/journal.pone.0253578)
Supplement: S2 Table — Undetermined is at 45 cycles of qRT-PCR. (DOCX) [file pone.0253578.s002.docx]

S2 Table

| **Sample number** | **Location** | **Object/Surface** | **CDC_N1** | **CDC_N2** |
| --- | --- | --- | --- | --- |
| 1 | Hospitalist room P2 | food table | Undetermined | Undetermined |
| 2 | Hospitalist room P2 | Keyboard | Undetermined | Undetermined |
| 3 | Hospitalist room P2 | conference table | Undetermined | Undetermined |
| 4 | Hospitalist room P2 | armrest (chair at conference table) | Undetermined | Undetermined |
| 5 | Residents' room T5 | Door knob/keypad, interior | Undetermined | Undetermined |
| 6 | Residents' room T5 | Keyboard | ND | ND |
| 7 | Residents' room T5 | microwave panel and door | Undetermined | Undetermined |
| 8 | Residents' room T5 | Telephone | Undetermined | Undetermined |
| 9 | Residents' room T5 | Keyboard | Undetermined | Undetermined |
| 10 | Residents' room D6 | Keyboard | Undetermined | Undetermined |
| 11 | Residents' room D6 | conference table | Undetermined | Undetermined |
| 12 | Residents' room D6 | Door knob/keypad, interior | Undetermined | Undetermined |
| 13 | Residents' room D6 | Telephone | Undetermined | Undetermined |
| 14 | Residents' room D6 | armrest (chair at conference table) AND "desktop" | Undetermined | Undetermined |
| 15 | Patient room D14 | bedrails (L) | ND | ND |
| 16 | Patient room D14 | bedrails (R) | Undetermined | Undetermined |
| 17 | Patient room D14 | IV pump console | Undetermined | Undetermined |
| 18 | Patient room D14 | 02 wall dial | Undetermined | Undetermined |
| 19 | Patient room D14 | marker/pen | Undetermined | Undetermined |
| 20 | Patient room D14 | Floor | **35.92** | **38.71** |
| 21 | Patient room D14 | thermometer handle | Undetermined | Undetermined |
| 22 | Patient room D14 | propac | Undetermined | Undetermined |
| 23 | Patient room D14 | mouse | Undetermined | Undetermined |
| 24 | Patient room D14 | Keyboard | Undetermined | Undetermined |
| 25 | Patient room D14 | rover | Undetermined | Undetermined |
| 26 | Patient room D14 | Ergotron | Undetermined | Undetermined |
| 27 | Patient room D14 | Floor | **32.09** | **33.72** |
| 28 | Patient room D14 | IV bag | Undetermined | Undetermined |
| 29 | Patient room D14 | Nightstand | Undetermined | Undetermined |
| 30 | Patient room D14 | hand sanitizer (in room) | Undetermined | Undetermined |
| 31 | Patient room D14 | Window | Undetermined | Undetermined |
| 32 | Patient room D14 | light switch | ND | ND |
| 33 | Patient room D14 | light switch | Undetermined | Undetermined |
| 34 | Patient room D14 | Door knob/keypad, interior | Undetermined | Undetermined |
| 35 | Patient room D14 | food table | Undetermined | Undetermined |
| 36 | Patient room D14 | isolation/PPE cart (room exterior) | Undetermined | Undetermined |
| 37 | Patient room T7 Blue | Window | Undetermined | Undetermined |
| 38 | Patient room T7 Blue | vent tubing arm | **36.83** | Undetermined |
| 39 | Patient room T7 Blue | Vent screen and knobs | Undetermined | Undetermined |
| 40 | Patient room T7 Blue | Keyboard | Undetermined | **37.62** |
| 41 | Patient room T7 Blue | Ergotron | Undetermined | Undetermined |
| 42 | Patient room T7 Blue | Floor | Undetermined | 43.06 |
| 43 | Patient room T7 Blue | Floor | Undetermined | Undetermined |
| 44 | Patient room T7 Blue | rover | Undetermined | Undetermined |
| 45 | Patient room T7 Blue | IV pump console | Undetermined | Undetermined |
| 46 | Patient room T7 Blue | Door knob/keypad, interior | Undetermined | Undetermined |
| 47 | Patient room T7 Blue | Nightstand | Undetermined | Undetermined |
| 48 | Patient room T7 Blue | telemetry screen alarm button | **37.07** | **38.6** |
| 49 | Patient room T7 Blue | Arterial line plunger | Undetermined | Undetermined |
| 50 | Patient room T7 Blue | pluerevac box handle | Undetermined | Undetermined |
| 51 | Patient room T7 Blue | soiled linen lid | **36.36** | **37.41** |
| 52 | Patient room T7 Blue | mouse | Undetermined | Undetermined |
| 53 | Patient room T7 Blue | CRRT console | Undetermined | Undetermined |
| 54 | Patient room T7 Blue | endotracheal tube straps | Undetermined | Undetermined |
| 55 | Patient room T7 Blue | bedrails (R) | ND | ND |
| 56 | Patient room T7 Blue | bedrails (L) | Undetermined | Undetermined |
| 57 | Patient room T7 Blue | marker/pen | Undetermined | Undetermined |
| 58 | Patient room T7 Blue | hand sanitizer (in room) | Undetermined | Undetermined |
| 59 | Patient room T7 Blue | doffing table (exterior, BD universal viral transport swab) | Undetermined | Undetermined |
| 60 | Patient room T7 Blue | sanitizer pump (exterior, BD Eswab for bacteria used) | Undetermined | Undetermined |
| ENV-1 | Floor Samples | Hand sanitizer dispenser | Undetermined | Undetermined |
| ENV-2 | Floor Samples | Sticker table screening area | Undetermined | Undetermined |
| ENV-3 | Floor Samples | Floor sample | Undetermined | Undetermined |
| ENV-4 | Floor Samples | Information desk counters middle window | Undetermined | Undetermined |
| ENV-5 | Floor Samples | Circular table between gift shop and bathroom | Undetermined | Undetermined |
| ENV-6 | Floor Samples | Water fountain in front of bathroom 1P154214 | Undetermined | Undetermined |
| ENV-7 | Floor Samples | Women's bathroom 1P154 | **39.3** | Undetermined |
| ENV-8 | Floor Samples | Women's bathroom 1P154 | Undetermined | Undetermined |
| ENV-9 | Floor Samples | Women's bathroom 1P154 | Undetermined | Undetermined |
| ENV-10 | Floor Samples | ATM in front of cardiovascular services | Undetermined | Undetermined |
| ENV-11 | Elevator Button | Elevator in front of cardiovascular services | Undetermined | Undetermined |
| ENV-12 | Elevator Button | Davis Tower, exterior elevator button, level 1 | Undetermined | Undetermined |
| ENV-13 | Elevator Button | Davis Tower, interior elevator button, right side | Undetermined | Undetermined |
| ENV-14 | Floor Samples | Patient transport wheelchairs | Undetermined | Undetermined |
| ENV-15 | Floor Samples | Patient transport wheelchairs | Undetermined | Undetermined |
| ENV-16 | Floor Samples | Information desk's mouse computer for self-serving PAVLNIO60 | Undetermined | Undetermined |
| ENV-17 | Floor Samples | Floor Sample | Undetermined | Undetermined |
| ENV-18 | Floor Samples | D14→ Intercom button by the elevators | Undetermined | Undetermined |
| ENV-19 | Offices of Ed, Pulm/ Crit Care Staff | Door handle | Undetermined | Undetermined |
| ENV-20 | Offices of Ed, Pulm/ Crit Care Staff | D10 intercom button for P1C4 | Undetermined | Undetermined |
| ENV-21 | Offices of Ed, Pulm/ Crit Care Staff | D10 Door handle for P1C4 | Undetermined | Undetermined |
| ENV-22 | Offices of Ed, Pulm/ Crit Care Staff | Outdoor cafeteria courtyard dining table | Undetermined | Undetermined |
| ENV-23 | Offices of Ed, Pulm/ Crit Care Staff | Outdoor cafeteria conference door chair arms | Undetermined | Undetermined |
| ENV-24 | Offices of Ed, Pulm/ Crit Care Staff | ER internal entrance door handle | Undetermined | Undetermined |
| ENV-25 | Offices of Ed, Pulm/ Crit Care Staff | ER "first nursing/ registration" counter | Undetermined | Undetermined |
| ENV-26 | Offices of Ed, Pulm/ Crit Care Staff | West Entrance- hand sanitizer dispenser | Undetermined | Undetermined |
| ENV-27 | Offices of Ed, Pulm/ Crit Care Staff | West Entrance Floor Sample | Undetermined | Undetermined |
| ENV-28 | Offices of Ed, Pulm/ Crit Care Staff | South Elevator- External buttons ↑↓ buttons 1st floor | Undetermined | Undetermined |
| ENV-29 | Offices of Ed, Pulm/ Crit Care Staff | Wellness Check counters at West Entrance | Undetermined | Undetermined |
| ENV-30 | Offices of Ed, Pulm/ Crit Care Staff | Investigational drug services pharmacy door handle | Undetermined | Undetermined |
| ENV-31 | Hallways | Stair 1 Floor 1 "subbasement to 8th floor" West Entrance/East Wing | Undetermined | Undetermined |
| ENV-32 | Hallways | Door handle facing North Addition | Undetermined | Undetermined |
| ENV-33 | Hallways | Hallway to North Addition floor sample middle | Undetermined | Undetermined |
| ENV-34 | Hallways | Handicap button to exit hospital | Undetermined | Undetermined |
| ENV-35 | Hallways | Handicap button to exit hospital | Undetermined | Undetermined |
| ENV-36 | Hallways | D8 Reception counter (Transplant Unit) eastxxx | Undetermined | Undetermined |
| ENV-37 | Hallways | Door handle of UT8→ ICU | Undetermined | Undetermined |
| ENV-38 | Hallways | University Tower elevators internal buttons | Undetermined | Undetermined |
| ENV-39 | Hallways | University Tower elevator external buttons | Undetermined | Undetermined |
| ENV-40 | Lab Space | Beckman Centrifuge (Left) | Undetermined | Undetermined |
| ENV-41 | Lab Space | Thermo Centrifuge (Mid) | Undetermined | Undetermined |
| ENV-42 | Lab Space | Eppendorf Centrifuge (Right) | Undetermined | Undetermined |
| ENV-43 | Lab Space | Freezer Handle | Undetermined | Undetermined |
| ENV-44 | Lab Space | Refrigerator Handle | Undetermined | Undetermined |
| ENV-45 | Lab Space | Infectious waste lid and foot pedal | Undetermined | Undetermined |
| ENV-46 | Lab Space | iPad | Undetermined | Undetermined |
| ENV-47 | Lab Space | pipettors | Undetermined | Undetermined |
| ENV-48 | Lab Space | Lab Bench | Undetermined | Undetermined |
| ENV-49 | Lab Space | Hand soap Handle | Undetermined | Undetermined |
| ENV-50 | Lab Space | Floor beneath lab area | Undetermined | Undetermined |
| ENV-51 | Lab Space | Bleach bottle | Undetermined | Undetermined |
| ENV-52 | Lab Space | Entrance door handle | Undetermined | Undetermined |
| ENV-53 | Research pt. room | Vitals equipment | Undetermined | Undetermined |
| ENV-54 | Research pt. room | Supplies door handle | Undetermined | Undetermined |
| ENV-55 | Research pt. room | Chair armrest (right) | Undetermined | Undetermined |
| ENV-56 | Research pt. room | Backrest | Undetermined | Undetermined |
| ENV-57 | Research pt. room | Hand soap | Undetermined | Undetermined |
| ENV-58 | Research pt. room | Infectious waste bin | Undetermined | Undetermined |
| ENV-59 | Research pt. room | Air vent? | Undetermined | Undetermined |
| ENV-60 | Research pt. room | Floor | Undetermined | Undetermined |
| ENV-61 | Research pt. room | DVD player buttons | Undetermined | Undetermined |
| ENV-62 | Research pt. room | Doorknob | Undetermined | Undetermined |
| ENV-63 | Research pt. room | Ethanol Spray Bottle | Undetermined | Undetermined |
| ENV-64 | Floor Sample | In front of South elevator | Undetermined | Undetermined |
| ENV-65 | Floor Sample | E6 entrance (by east elevator entrance) | Undetermined | Undetermined |
| ENV-66 | Door Handle | E6 entrance | Undetermined | Undetermined |
| ENV-67 | Elevator Button | Middle Elevator/Buttons | Undetermined | Undetermined |
| ENV-68 | Floor Sample | Side by 6004 | Undetermined | Undetermined |
| ENV-69 | Door Handle | E5 double door | Undetermined | Undetermined |
| ENV-70 | Floor Sample | Double door | Undetermined | Undetermined |
| ENV-71 | XXX | Single door entry to patient rooms (E5) | Undetermined | Undetermined |
| ENV-72 | Door Handle | South Wing Level 3 | Undetermined | Undetermined |
| ENV-73 | Floor Sample | In front of room 3005 | Undetermined | Undetermined |
| ENV-74 | Floor Sample | In front of level 3 | Undetermined | Undetermined |
| ENV-75 | Greeter | Front Entry Badge | Undetermined | Undetermined |
| ENV-76 | Greeter | Front Entry Badge | Undetermined | Undetermined |
| ENV-77 | Greeter | Front Entry Badge | Undetermined | Undetermined |
| ENV-78 | Divider | Pharmacy Divider/counter | Undetermined | Undetermined |
| ENV-79 | Divider | Pharmacy Divider/counter ("other side") | Undetermined | Undetermined |
| ENV-80 | Divider | Security Main Entrance/ Divider | Undetermined | Undetermined |
| ENV-81 | ER | ER wellness cheek | Undetermined | Undetermined |
| ENV-82 | ER | Greeter RN badge/ ER entry | Undetermined | Undetermined |
| ENV-83 | Divider | Divider in ER main entry | Undetermined | Undetermined |
| ENV-84 | Divider | Divider in ER registration desk | Undetermined | Undetermined |
| ENV-85 | Entrance Door | Hand Sanitizer dispenser | Undetermined | Undetermined |
| ENV-86 | ER | PA registration Rep Badge | Undetermined | Undetermined |
| ENV-87 | ER | ER wheelchair handle | Undetermined | Undetermined |
| ENV-88 | ER | ER wheelchair patient arm rest | Undetermined | Undetermined |
| ENV-89 |  | courtyard table | Undetermined | Undetermined |
| ENV-90 |  | courtyard table divider | Undetermined | Undetermined |
| ENV-91 |  | Staff Badge: Pharmacist Resident | Undetermined | Undetermined |
| ENV-92 |  | Vocera: Staff pharmacist | Undetermined | Undetermined |
| ENV-93 | ICU Patient- COVID+ | Floor- Patient Left | Undetermined | Undetermined |
| ENV-94 | ICU Patient- COVID+ | Floor- Patient Right | Undetermined | Undetermined |
| ENV-95 | ICU Patient- COVID+ | Floor- Near Door | Undetermined | Undetermined |
| ENV-96 | ICU Patient- COVID+ | Ventilator Tubing Intake | Undetermined | Undetermined |
| ENV-97 | ICU Patient- COVID+ | Ventilator Tubing Outflow | Undetermined | Undetermined |
| ENV-98 | ICU Patient- COVID+ | Ventilator | Undetermined | Undetermined |
| ENV-99 | ICU Patient- COVID+ | Linen Cart- pedal | Undetermined | Undetermined |
| ENV-100 | ICU Patient- COVID+ | Linen Cart- lid | **35.16** | **37.27** |
| ENV-101 | ICU Patient- COVID+ | Bedside Table | Undetermined | Undetermined |
| ENV-102 | ICU Patient- COVID+ | Bedrail- Left Lower | Undetermined | Undetermined |
| ENV-103 | ICU Patient- COVID+ | Bedrail- Left Upper | Undetermined | Undetermined |
| ENV-104 | ICU Patient- COVID+ | Bedrail- Right Lower | Undetermined | Undetermined |
| ENV-105 | ICU Patient- COVID+ | Infusion Pump | Undetermined | Undetermined |
| ENV-106 | ICU Patient- COVID+ | Biohazard Bins | Undetermined | Undetermined |
| ENV-107 | ICU Patient- COVID+ | Hand sanitizer dispenser- near doorway | Undetermined | Undetermined |
| ENV-108 | ICU Patient- COVID+ | Room Door Handle | Undetermined | **41.91** |
| ENV-109 | ICU Patient- COVID+ | Nurse Rover Device | Undetermined | Undetermined |
| ENV-110 | ICU Patient- COVID+ | Bedside Buttons- Left | Undetermined | Undetermined |
| ENV-111 | ICU Patient- COVID+ | Whiteboard markers | Undetermined | Undetermined |
| ENV-112 | ICU Patient- COVID+ | Light Switches | Undetermined | Undetermined |
| ENV-113 | Floor Neighboring COVID+ Floor patient | Floor- Foot of bed | Undetermined | Undetermined |
| ENV-114 | Floor Neighboring COVID+ Floor patient | Floor- Right of bed | Undetermined | Undetermined |
| ENV-115 | Floor Neighboring COVID+ Floor patient | Floor- Left of bed | Undetermined | Undetermined |
| ENV-116 | Floor Neighboring COVID+ Floor patient | Floor- Bathroom door | Undetermined | Undetermined |
| ENV-117 | Floor Neighboring COVID+ Floor patient | Floor- Main room door | Undetermined | Undetermined |
| ENV-118 | Floor Neighboring COVID+ Floor patient | Handle- Bathroom door | Undetermined | Undetermined |
| ENV-119 | Floor Neighboring COVID+ Floor patient | Floor- Bathroom floor | Undetermined | Undetermined |
| ENV-120 | Floor Neighboring COVID+ Floor patient | Sink- Bathroom | Undetermined | Undetermined |
| ENV-121 | Floor Neighboring COVID+ Floor patient | Toilet | Undetermined | Undetermined |
| ENV-122 | Floor Neighboring COVID+ Floor patient | Workstation Keyboard (in room) | Undetermined | Undetermined |
| ENV-123 | Floor Neighboring COVID+ Floor patient | Computer monitor | Undetermined | Undetermined |
| ENV-124 | Floor Neighboring COVID+ Floor patient | Linen cart | Undetermined | Undetermined |
| ENV-125 | Floor Neighboring COVID+ Floor patient | Trash bin | Undetermined | Undetermined |
| ENV-126 | Floor Neighboring COVID+ Floor patient | Handle- Main room door | Undetermined | Undetermined |
| ENV-127 | Floor Neighboring COVID+ Floor patient | Hand sanitizer dispenser- near room sink | Undetermined | Undetermined |
| ENV-128 | Floor Neighboring COVID+ Floor patient | Sink- in room sink pedals | Undetermined | Undetermined |
| ENV-129 | Floor Neighboring COVID+ Floor patient | Whiteboard markers | Undetermined | Undetermined |
| ENV-130 | Floor Neighboring COVID+ Floor patient | Sharps lid and container | Undetermined | Undetermined |
| ENV-131 | Floor Neighboring COVID+ Floor patient | Bedrail- left side | Undetermined | Undetermined |
| ENV-132 | Floor Neighboring COVID+ Floor patient | Bedside Buttons- Left | Undetermined | Undetermined |
| ENV-133 | Floor Neighboring COVID+ Floor patient | Bedside Table | Undetermined | Undetermined |
| ENV-134 | Floor Neighboring COVID+ Floor patient | O2 Flow Regulator knob | Undetermined | Undetermined |
| ENV-135 | Floor Neighboring COVID+ Floor patient | Device Plug | Undetermined | Undetermined |
| ENV-136 | Floor Neighboring COVID+ Floor patient | Stethoscope (in room) | Undetermined | Undetermined |
| ENV-137 | Floor Nursing Workspace | Floor- Nursing Workspace | **37.97** | Undetermined |
| ENV-138 | Floor Nursing Workspace | Nurse Desk | Undetermined | Undetermined |
| ENV-139 | Floor Nursing Workspace | Nurse Sink | Undetermined | Undetermined |
| ENV-140 | Floor Nursing Workspace | Nurse Phone | Undetermined | Undetermined |
| ENV-141 | Floor Nursing Workspace | Rover in Nurse Space | Undetermined | Undetermined |
| ENV-142 | Floor Nursing Workspace | Nurse's Vocera (who has been in COVID+ patient room) | Undetermined | Undetermined |
| ENV-143 | Floor Nursing Workspace | Nurse's Badge (who has been in COVID+ patient room) | Undetermined | Undetermined |
| ENV-144 | Floor Patient- COVID+ | Floor- Left of bed | **43.73** | Undetermined |
| ENV-145 | Floor Patient- COVID+ | Floor- Foot of bed | Undetermined | Undetermined |
| ENV-146 | Floor Patient- COVID+ | Floor- Right of bed | Undetermined | Undetermined |
| ENV-147 | Floor Patient- COVID+ | Workstation Keyboard (in room) | Undetermined | Undetermined |
| ENV-148 | Floor Patient- COVID+ | Workstation Desk Surface | Undetermined | Undetermined |
| ENV-149 | Floor Patient- COVID+ | Floor- Bathroom | Undetermined | Undetermined |
| ENV-150 | Floor Patient- COVID+ | Handle- Bathroom door | Undetermined | Undetermined |
| ENV-151 | Floor Patient- COVID+ | Sink- Bathroom | Undetermined | Undetermined |
| ENV-152 | Floor Patient- COVID+ | Toilet | Undetermined | Undetermined |
| ENV-153 | Floor Patient- COVID+ | Bedrail- Left | Undetermined | Undetermined |
| ENV-154 | Floor Patient- COVID+ | Bedrail- Right | Undetermined | Undetermined |
| ENV-155 | Floor Patient- COVID+ | Infusion Pump | Undetermined | Undetermined |
| ENV-156 | Floor Patient- COVID+ | Bedside Table | Undetermined | Undetermined |
| ENV-157 | Floor Patient- COVID+ | O2 Flow Regulator knob | Undetermined | Undetermined |
| ENV-158 | Floor Patient- COVID+ | Trash bin | Undetermined | Undetermined |
| ENV-159 | Floor Patient- COVID+ | Handle- Main room door | Undetermined | Undetermined |
| ENV-160 | Floor Patient- COVID+ | Bedside Buttons- Left | Undetermined | Undetermined |
| ENV-161 | Floor Patient- COVID+ | Hand sanitizer dispenser | Undetermined | Undetermined |
| ENV-162 | Floor Patient- COVID+ | Stethoscope (in room) | Undetermined | Undetermined |
| ENV-163 | Floor Patient- COVID+ | Linen Cart | Undetermined | Undetermined |
| ENV-164 | Floor Patient- COVID+ | Sharps lid and container | Undetermined | Undetermined |
| ENV-165 | Floor Patient- COVID+ | Patient Call Light | Undetermined | Undetermined |
| ENV-166 | Floor Patient- COVID+ | Device Plug | Undetermined | Undetermined |
| ENV-167 | Floor Patient- COVID+ | Thermometer | Undetermined | Undetermined |
| ENV-168 | Floor Patient- COVID+ | Sink- Antechamber Pedals | Undetermined | Undetermined |
