# Supplementary material for: SARS-CoV-2 detection and genomic sequencing from hospital surface samples collected at UC Davis
Source: PLoS One. 2021 Jun 24;16(6):e0253578. doi: 10.1371/journal.pone.0253578 (PMC8224861; doi:10.1371/journal.pone.0253578)
Supplement: S3 Table — (DOCX) [file pone.0253578.s003.docx]

S3 Table

| **Run Number** | **Total Reads** | **Barcoded**  **Reads** | **Unclassified Reads** | **Quality Controlled Reads** | **Percent Reads Passing QC** |
| --- | --- | --- | --- | --- | --- |
| Run 1 | 405,968 | 142,204 | 263,765 | 63,048 | 15.53 |
| Run 2 | 14,804,576 | 8,694,698 | 6,109,879 | 6,404,977 | 43.26 |
| Run 3 | 1,576,082 | 996,781 | 579,302 | 50,420 | 3.2 |
| Run 4 | 1,223 | 341 | 883 | 19 | 1.55 |
| Run 5 | 780,000 | 232,771 | 547,230 | 152,152 | 19.51 |
