# Supplementary material for: SARS-CoV-2 detection and genomic sequencing from hospital surface samples collected at UC Davis
Source: PLoS One. 2021 Jun 24;16(6):e0253578. doi: 10.1371/journal.pone.0253578 (PMC8224861; doi:10.1371/journal.pone.0253578)
Supplement: Attachment — Submitted filename: Response to Reviewers.docx [file pone.0253578.s004.docx]

Dear Dr. Kumar,

We are submitting here the revised version of our manuscript “SARS-CoV-2 detection and genomic sequencing from hospital surface samples collected at UC Davis”. We thank the editor and reviewers for their detailed comments and suggestions and have made revisions in response to these.

*Reviewer #1: The Manuscript by Coil et al deals with an important issue in the current pandemic situation whether different hospital surfaces possess contagious and infective SARS-Cov2 viral particles. The study clearly showed that although viral genome is found in many surfaces in hospital setting but they are not contagious at all. The authors also identified that the important issue related to proper cleaning management helps to reduce RT-PCR positivity in different hospital surfaces. Using whole genome sequencing authors recovered a near-complete viral genome from various surface swabs.*

*Overall the work is impressive with few minor concerns listed below:*

*1. While constructing the phylogenetic tree, the authors claim that the samples where near-complete genome was recovered by sequencing were derived from single patient. This might also be possible that multiple patients were infected with similar virus since at the time of study only few variants/mutants were in USA/UCDMC. This statement needs to be corrected both in results section (line – 289) and conclusion (line 301-302) section. That might be more correct scientific way to discuss the issue.*

Please, see the statement that multiple patients could have been infected with very similar viruses in the Results section: “...Thus, it appears likely these samples were derived from a single patient (or from multiple patients infected with similar viruses) from which USA/CA-CZB-1145/2020 originated...” We have also updated the Conclusion section with similar language.

*2. There are couple of reports in others study also showed that there is minimal chance of viral infectivity from different surfaces. There are also several reports suggest that cleaning reduces viral particles on surfaces. Thus this manuscript lacks novelty in this perspective.*

We have cited the major studies in the Introduction that support that surfaces present a minimal risk of transmission and the significance of hospital ICU cleaning practices and we acknowledge that our study provides support to these important findings.

*3. The conclusion section needs to be elaborated with reference to previous findings in this context and a brief necessary discussion will help readers to better understand the importance of this work.*

We have referenced numerous other studies that have looked at surfaces and cleaning (with or without infectivity data) in the introduction and we have modified the language to clarify this. The focus of the conclusions is on the major novel findings in our work stating that viral gene sequencing is possible from samples obtained from surfaces and that sequencing may be more sensitive than qRT-PCR in detecting viral RNA.

*4. Figure Legends section – Figure 5 heading needs to be bold character.*

We fixed this

*Reviewer #2: The manuscript by Coil et. al. aims to study the surface swab of SARS-COV2- RNA and determine its applicability in sequencing studies. Overall, this is a well written manuscript which looks at an extremely relevant topic and will add to the existing knowledge about the COVID-19 pandemic. Furthermore, this study has the potential to be informative in cases where a new cleaning method is being tested in places such as long-term senior care facilities as we all to evaluate the effectiveness of different control measures. Although the study provides evidence of low likelihood that hospital surface samples (with proper cleaning method in place) contains low likelihood of active transmission the article can include in the conclusion that the results should not encourage to replace the existing public health measures of keeping good hygiene and physical distancing to prevent transmission.*

We have added a sentence to the Conclusions highlighting this point.

*Additionally, the study should mention in the discussion that the conclusion should be considered keeping the following in mind that it was conducted in just one medical center at a specific humidity, temperature, UV light, ventilation, occupancy, activity level and environmental context and importantly at a time when the different variants of SARS-COV2 that we see currently were not known.*

We have now included this clarification in the Discussion

*Please also mention the for which gene the RT-PCR were conducted in the main text. Overall, this is a relevant and well written manuscript.*

We have added this information to the Results section on line 187. And thank you.
